# Exome sequencing in individuals with congenital anomalies of the kidney and urinary tract (CAKUT): a single-center experience

**DOI:** 10.1038/s41431-023-01331-x

**Published:** 2023-03-16

**Authors:** Korbinian M. Riedhammer, Jasmina Ćomić, Velibor Tasic, Jovana Putnik, Nora Abazi-Emini, Aleksandra Paripovic, Natasa Stajic, Thomas Meitinger, Valbona Nushi-Stavileci, Riccardo Berutti, Matthias C. Braunisch, Julia Hoefele

**Affiliations:** 1 Institute of Human Genetics, Klinikum rechts der Isar, Technical University of Munich, School of Medicine, Munich, Germany; 2 Department of Nephrology, Klinikum rechts der Isar, Technical University of Munich, School of Medicine, Munich, Germany; 3 University Children’s Hospital, Medical Faculty of Skopje, Skopje, North Macedonia; 4 Institute for Mother and Child Health Care of Serbia “Dr Vukan Čupić”, Department of Nephrology, University of Belgrade, Faculty of Medicine, Belgrade, Serbia; 5 Pediatric Clinic, University Clinical Center of Kosovo, Prishtina, Kosovo

**Keywords:** Paediatric kidney disease, Genetic counselling

## Abstract

Individuals with congenital anomalies of the kidney and urinary tract (CAKUT) show a broad spectrum of malformations. CAKUT can occur in an isolated fashion or as part of a syndromic disorder and can lead to end-stage kidney failure. A monogenic cause can be identified in ~12% of affected individuals. This study investigated a single-center CAKUT cohort analyzed by exome sequencing (ES). Emphasis was placed on the question whether diagnostic yield differs between certain CAKUT phenotypes (e.g., bilateral kidney affection, unilateral kidney affection or only urinary tract affection). 86 unrelated individuals with CAKUT were categorized according to their phenotype and analyzed by ES to identify a monogenic cause. Prioritized variants were rated according to the recommendations of the American College of Medical Genetics and Genomics and the Association for Clinical Genomic Science. Diagnostic yields of different phenotypic categories were compared. Clinical data were collected using a standardized questionnaire. In the study cohort, 7/86 individuals had a (likely) pathogenic variant in the genes *PAX2*, *PBX1*, *EYA1*, or *SALL1*. Additionally, in one individual, a 17q12 deletion syndrome (including *HNF1B*) was detected. 64 individuals had a kidney affection, which was bilateral in 36. All solved cases (8/86, 9%) had bilateral kidney affection (diagnostic yield in subcohort: 8/36, 22%). Although the diagnostic yield in CAKUT cohorts is low, our single-center experience argues, that, in individuals with bilateral kidney affection, monogenic burden is higher than in those with unilateral kidney or only urinary tract affection.

## Introduction

Congenital anomalies of the kidney and urinary tract (CAKUT) is a collective term for a group of diverse structural malformations ranging from relatively mild phenotypes like vesicoureteral reflux (VUR) to severe manifestations like bilateral renal agenesis. CAKUT can be present in isolated form (limited to the kidney and the urinary tract) or can be part of syndromic disorders [1]. It results from disturbances in embryonic development of the kidney and/or the urinary tract and is the leading cause for end-stage kidney failure in the pediatric population. CAKUT accounts for 23% of all birth defects and has a prevalence of about 3–6 per 1000 newborns [2–6].

Perturbation of renal and ureteral morphogenesis in CAKUT can be due to environmental, epigenetic, genetic factors, and the interplay of these [2]. Although alterations in renal development can be multifactorial, there are also hereditary forms of CAKUT resulting from single-gene defects (“monogenic CAKUT”). This is underscored by familial clustering of CAKUT, knockout mouse models recapitulating CAKUT phenotypes, and complex heritable syndromes involving CAKUT [1, 7]. Hence, it is not surprising that disease-causing variants in more than 50 different genes and with different modes of inheritance (e.g., autosomal dominant, autosomal recessive, X-linked) have been described in the literature [8]. Additionally, numerical chromosomal alterations, deletions or duplications are known to cause CAKUT [9, 10].

However, in only about 16% of individuals affected by CAKUT, a monogenic cause can be identified, with diagnostic yields varying between 6% and 33% depending on study population. It has been shown that the diagnostic yield is higher if there is familial or syndromic occurrence of CAKUT [11]. Furthermore, there is some evidence that individuals with a severe CAKUT phenotype, such as renal agenesis or renal dysplasia, have a higher diagnostic yield (17% in one publication) but more detailed data on the relationship between phenotypic variation (e.g., bilateral vs. unilateral vs. only urinary tract affection) and the respective diagnostic yield in CAKUT is lacking [6, 12].

Molecular genetic techniques like next-generation sequencing including exome sequencing (ES), linkage analyses, homozygosity mapping combined with ES for recessive diseases, and array analysis are the current approaches to identify disease-causing variants in known disease-associated CAKUT genes, but also to discover new candidate genes [2, 8, 13–15]. This study investigated a tertiary care center CAKUT cohort analyzed by ES. Focus was specifically laid on whether diagnostic yield is different in certain CAKUT phenotypes (e.g., bilateral kidney affection, unilateral kidney affection or only urinary tract affection).

## Material and methods

### Study population

**Fig. 1:**
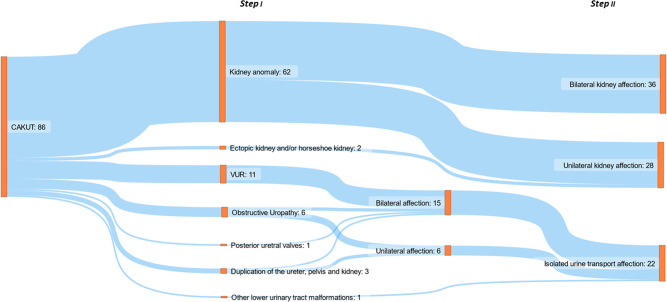
Cohort overview according to renal and urinary tract phenotype (two-step process, see Methods section). This figure was created with the free web-based tool SankeyMATIC (http://sankeymatic.com/build/). CAKUT congenital anomalies of the kidney and urinary tract, VUR vesicoureteral reflux.

A cohort of 86 unrelated index cases with a CAKUT phenotype was collected at our institute between October 2015 and February 2019 after referral by their clinician (Fig. 1). This study was performed according to the standards of the Declaration of Helsinki 2013 and approved by the local Ethics Committee of the Technical University of Munich (approval number 521/16S). Written informed consent was obtained from all individuals or their legal guardians. Clinical and phenotypic data were obtained from clinical reports and medical history. A standardized questionnaire was used to assess the clinical information.

Each individual was assigned to one group each in a two-step process:

First, all individuals were categorized into one of the following groups based on their renal/urinary tract phenotype (according to ref. [16]). These groups are as follows: group I: duplication of the ureter, pelvis and kidney; group II: ectopic kidney and/or horseshoe kidney; group III: other kidney anomaly; group IV: obstructive uropathy; group V: VUR; group VI: posterior ureteral valves; and group VII: other lower urinary tract malformations.

Secondly, the individuals were divided into the following three groups encompassing a broader phenotypic spectrum; group I: “bilateral kidney affection”, in which cases with any bilateral renal affection were included; group II: “unilateral kidney affection”, in which unilateral kidney affection and cases with unilateral ectopic kidney and/or a horseshoe kidney were included; group III: “isolated urine transport affection” (i.e., without known parenchymal kidney affection), in which cases with duplication of the ureter, pelvis and kidney, obstructive uropathy, posterior ureteral valves, VUR and other lower urinary tract malformations phenotypes were included.

### Genetic testing

Peripheral blood was used for DNA isolation using the automated nucleic acid purification instrument Chemagic™ 360 (PerkinElmer, Waltham, MA, USA) according to the manufacturer’s protocol.

### Exome sequencing

ES was performed with Sure Select Human All Exon V5 (50 Mb) Kit (Agilent Technologies, Inc., Santa Clara, CA, United States of America) and a HiSeq2500 (Illumina, Inc., San Diego, CA, United States of America) or with Sure Select Human All Exon V6 (60 Mb) Kit (Agilent Technologies, Inc., Santa Clara, CA, United States of America) and a HiSeq4000 (Illumina, Inc., San Diego, CA, United States of America) [17]. Mitochondrial DNA was derived from off-target exome reads as previously described [18]. Reads were aligned to the Genome Reference Consortium Human Build 37 (UCSC Genome Browser build hg19) using Burrows-Wheeler Aligner (v.0.7.5a). SAMtools (version 0.1.19) was employed for detection of single-nucleotide variants (SNVs) and small insertions and deletions (indels). ExomeDepth was used to detect copy number variations (CNVs). A noise threshold of 2.5 was accepted for diagnostic analysis [19]. The retrieved CNVs were visualized using Integrative Genomics Viewer (IGV, https://software.broadinstitute.org/software/igv/) to verify that the regions examined were adequately covered and that the CNVs were plausible. CNVs were then compared with publicly available control databases like the Genome Aggregation Database (gnomAD, https://gnomad.broadinstitute.org/about), the Database of Genomic Variants (DGV, http://dgv.tcag.ca/dgv/app/home), and databases for disease-causing CNVs like DECIPHER (https://decipher.sanger.ac.uk/) and ClinVar (https://www.ncbi.nlm.nih.gov/clinvar/).

### Sanger sequencing

Segregation analysis of a variant previously identified with ES was performed by Sanger sequencing in the parents to determine inheritance or to confirm a de novo status (if parents had not been exome sequenced in the first place). The oligonucleotide primer sequences are available upon request.

### Variant interpretation

For the analysis of de novo, autosomal dominant and mitochondrial variants, only variants with a minor allele frequency (MAF) of less than 0.1% in the in-house database of the Helmholtz Zentrum Munich containing more than 20,000 exomes were considered. For the analysis of autosomal recessive and X-linked variants (homozygous, hemizygous or [putative] compound heterozygous), variants with a MAF of less than 1.0% and a SNV quality of 30 were considered [20, 21]. Variants were checked in publicly available databases for (likely) pathogenic variants. These databases were ClinVar (https://www.ncbi.nlm.nih.gov/clinvar/), the Human Gene Mutation Database (HGMD^®^ Professional, http://www.hgmd.cf.ac.uk), and the Leiden Open Variation Database (LOVD, https://www.lovd.nl). The variants were rated in accordance with the guidelines of the American College of Medical Genetics (ACMG) and current amendments [22–25]. Individuals with a (likely) pathogenic variant and a fitting genotype were classified as “solved cases” or, if no disease-causing (i.e., likely pathogenic or pathogenic) variant could be identified, we designated them as “unsolved cases”.

## Results

### Study population

The total study cohort consisted of 86 index cases, 29/86 (34%) were female and 57/86 (66%) were male (Table 1). ES was performed in all index cases, 51 individuals were analyzed by proband-only ES, one by duo ES (index case and mother) and 34 by trio ES (index case and parents). Most of these cases were of non-Finnish origin (74/86; 86%; Germany, Serbia, and North Macedonia). 15/86 (17%) cases had a positive family history for CAKUT. Among those with reported parental consanguinity (7/86; 8%), family history was positive in 2/7 (29%) cases, and among those with no parental consanguinity (79/86; 92%), family history was positive in 13/79 (16%) cases. The median age at ES was 9 years (range 0 to 61 years). Extrarenal manifestations (syndromic CAKUT) were present in 33/86 (38%) cases and complex CAKUT (i.e., several CAKUT manifestations in an individual) in 24/86 (28%) cases (Table 1).

**Table 1:** Clinical characteristics of the 86 index cases.

Clinical characteristics	Total cohort (*n* = 86 index cases)
Sex	
Female	29 (34%)
Male	57 (66%)
Non-Finnish European descent	
Yes	74 (86%)
No	12 (14%)
Reported family history regarding CAKUT	Familial CAKUT
Yes	15 (17%)
No	71 (83%)
Reported parental consanguinity	
Yes	7 (8%)
No	79 (92%)
Syndromic CAKUT	
Yes	33 (38%)
No	53 (62%)
Syndromic features	
Skeletal malformations	9 (27%)
Eye anomalies	3 (9%)
Hearing impairment	3 (9%)
Intellectual disability	6 (18%)
Heart defect	6 (18%)
Complex CAKUT	
Yes	24 (30%)
No	62 (72%)
CAKUT phenotype	
Bilateral kidney affection	36 (42%)
Unilateral kidney affection	28 (33%)
Isolated urine transport affection	22 (25%)
Solved cases*	
Yes	8 (9%)
No	78 (91%)

*CAKUT* congenital anomalies of the kidney and urinary tract; *Solved cases are those in which a genetic diagnosis could be made by exome sequencing.

### All individuals were classified using a two-step process

#### Step I: Detailed renal and urinary tract phenotype

All 86 individuals were categorized into groups based on their renal and urinary tract phenotype (as per [16]; Fig. 1): group I: duplication of the ureter, pelvis and kidney: 3/86 (4%) cases; group II: ectopic kidney and/or horseshoe kidney: 2/86 (2%) cases; group III: other kidney anomaly: 62/86 (72%) cases; group IV: obstructive uropathy: 6/86 (7%) cases; group V: VUR: 11/86 (13%) cases: 11/86 (13%) cases; group VI: posterior ureteral valves: 1/86 (1%) cases; group VII: other lower urinary tract malformations: 1/86 (1%) cases.

#### Step II: Broader phenotype - bilateral or unilateral kidney affection or isolated urine transport affection

Grouping the individuals according to extent of affection, the following distribution was seen: group I: “bilateral kidney affection”: 36/86 (42%) cases; group II: “unilateral kidney affection”: 28/86 (32%) cases; group III: “isolated urine transport affection”: 22/86 (26%) cases (Fig. 1, Table 1).

#### Diagnostic yield

**Fig. 2:**
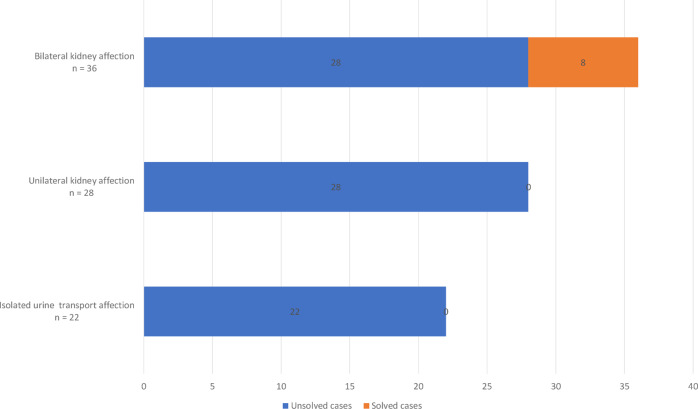
Distribution of solved cases in the CAKUT cohort consisting of 86 index cases grouped according to broader phenotype categories. Solved cases are those in which a genetic diagnosis could be made by exome sequencing.

Monogenic causes of CAKUT could be identified in 8/86 (9%) cases (Table 2 and Supplementary Table 1). All solved cases were in the “bilateral kidney affection” group (8/36; 22%; Fig. 2). 6/8 (75%) solved cases additionally had extrarenal manifestations (i.e., syndromic CAKUT).

**Table 2:** Phenotypic and genotypic details on genetically solved index cases in the cohort.

ID	Sex	Phenotype	Gene (transcript)	Chromosomal position (hg19)	Nucleotide and amino acid change	gnomAD v.2.1.1 MAF^*^	Zygosity	Inheritance	Genetic diagnosis (MIM phenotype number)**	Classification according to applied ACMG criteria/CNV score^***^	Accession number in ClinVar****	Individual ID in LOVD^#^	Syndromic disease (0 = no; 1 = yes)
HN-F262-II-1	f	Bilateral renal hypoplasia	*PAX2* (NM_000278.3)	chr10:g.102509535dup	c.76dup p.(Val26Glyfs*28)	0.00002834	Heterozygous	Father	Papillorenal syndrome (#120330)	Likely pathogenic (PVS1 PS4_moderate)	SCV001149865.1	-	0
HN-F75-II-1	m	Bilateral renal dysplasia, developmental delay, growth retardation, skeletal anomalies	*PBX1* (NM_002585.3)	chr1:g.164761878_164761884del	c.413_419del p.(Gly138Valfs*40)	Not listed	Heterozygous	de novo	CAKUTHED (#617641)	Pathogenic (PVS1 PS2 PM2)	SCV000680322.1	-	1
HN-F317-II-3	f	Bilateral renal agenesis, skeletal anomalies	*EYA1* (NM_000503.4)	chr8:g.72211430G>T	c.678C>A p.(Tyr226*)	Not listed	Heterozygous	de novo	Branchiootorenal syndrome 1, with or without cataracts (#113650)	Pathogenic (PVS1 PS2 PM2)	SCV001149770.1	-	1
HN-F541-II-2	f	Bilateral renal hypoplasia (cysts in right kidney), urogenital malformations, dysplastic ears, bilateral hexadactyly	*SALL1* (NM_002968.2)	chr16:g.51175307G>A	c.826C>T p.(Arg276*)	Not listed	Heterozygous	de novo	Townes-Brocks syndrome 1 (#107480)	Pathogenic (PVS1 PS2 PS4_moderate PM2)	SCV001430043.1	-	1
HN-F197-II-1	f	Bilateral renal cystic dysplasia	chr17q12 (-)	Approx. chr17:g.34842543-36104875	1.2 Mb deletion (including *HNF1B*)	0.0001393	Heterozygous	de novo	Chromosome 17q12 deletion syndrome (#614527)	Pathogenic (1,00)	-	00404564	0
HN-F305-II-1	m	Right renal agenesis, left renal hypoplasia, short stature, facial dysmorphies, choanal atresia	*PBX1* (NM_002585.3)	chr1:g.164769086G>T	c.661G>T p.(Glu221*)	Not listed	Heterozygous	de novo	CAKUTHED (#617641)	Pathogenic (PVS1 PS2 PM2)	SCV001149866.1	-	1
HN-F502-II-1	m	Bilateral renal hypoplasia, imperforate anus, dysplastic ears, right triphalangeal thumb	*SALL1* (NM_002968.2)	chr16:g.51173444_51173447dup	c.2686_2689dup p.(Val897Glyfs*6)	Not listed	Heterozygous	Undetermined	Townes-Brocks syndrome 1(#107480)	Likely pathogenic (PVS1 PM2)	SCV001149911.1	-	1
HN-F316-I-1	m	Branchiootorenal syndrome, congenital hypertrophic cardiomyopathy	*EYA1* (NM_000503.4)	chr8:g.72127964C>T	c.1361-1G>A p.(?)	Not listed	Heterozygous	Undetermined	Branchiootorenal syndrome 1, with or without cataracts (#113650)	Likely pathogenic (PVS1 PM2)	SCV001149769.1	-	1

*CAKUT* congenital anomalies of the kidney and urinary tract; *f* female; *m* male; *https://gnomad.broadinstitute.org/; **https://www.omim.org/; ***variant is classified as likely pathogenic/pathogenic as per ACMG and amendments [24, 25]; ****https://www.ncbi.nlm.nih.gov/clinvar/; ^#^https://www.lovd.nl.

Of the 22 complex CAKUT cases, only one (5%) case was solved. This case (HN-F541-II-2) presented with bilateral kidney hypoplasia, urogenital malformations, dysplastic ears, and bilateral hexadactyly (Tables 1, 2). 3/8 (38%) solved cases had a positive family history (HN-F262-II-1, HN-F502-II-1 and HN-F316-I-1). In all solved cases, there was no parental consanguinity (Table 2).

Of the 8 solved cases (Table 2), heterozygous disease-causing variants in *PBX1* were identified in 2/8 (25%) cases compatible with the genetic diagnosis of CAKUTHED (MIM #617641). In 2/8 (25%) cases, Townes-Brocks syndrome 1 (MIM #107480) was diagnosed, with one pathogenic and one likely pathogenic variant in *SALL1*. In 2/8 (25%) cases, Branchiootorenal syndrome 1 (MIM # 113650) was diagnosed, with a likely pathogenic and a pathogenic variant in *EYA1*. 1/8 (13%) cases had a papillorenal syndrome (MIM #120330) caused by a likely pathogenic variant in *PAX2*. Additionally, in 1/8 (13%) cases, a chromosome 17q12 deletion syndrome [MIM #614527] was detected which included *HNF1B*. 5/8 (63%) disease-causing variants have not been described in the literature so far. Of note, 6/8 (75%) of solved cases also had syndromic features (Table 2).

Supplementary Table 2 lists homozygous stretches in exome sequencing data and homozygous rare variants (only variants in OMIM-listed [https://www.omim.org/] disease-associated genes; MAF < 1.0%) within these stretches in consanguineous cases (all unsolved). Supplementary Table 3 lists rare de novo variants (MAF < 0.1%; CADD [https://cadd.gs.washington.edu/] >15) and rare homozygous/compound heterozygous/hemizygous variants (MAF < 1.0%) in unsolved trio ES cases (also only variants in OMIM-listed disease-associated genes).

### Diagnostic yields in different CAKUT groups

All solved cases had a bilateral kidney affection. No disease-causing variant could be found in individuals with a unilateral renal or an isolated urinary tract phenotype.

## Discussion

### Diagnostic yield

In this single-center cohort comprising 86 unrelated individuals, comprehensive genetic testing with ES resulted in a diagnostic yield of monogenic CAKUT of 9%. This is comparable with the reported diagnostics yields of unselected CAKUT cohorts in the literature. For example, in the study by van der Ven et al. 2018, in 29/232 (13%) families, disease-causing variants in genes associated with monogenic CAKUT were identified [6]. All eight solved cases of our cohort had bilateral kidney affection (8/36 individuals, diagnostic yield of 22% in this subcohort). Interestingly, in the above-mentioned study by van der Ven et al., cases with kidney involvement (renal agenesis or renal dysplasia; defined as “severe” CAKUT) also had a higher diagnostic yield of 17%. Discrepancies could be explainable by different compositions of the study cohorts, e.g., based on different inclusion criteria and the variability of the clinical phenotypes of the affected individuals. Of note, a precise differentiation between the diagnostic yields of unilateral and bilateral affections was not made in the study by van der Ven et al. Bekheirnia et al. 2017 identified pathogenic SNVs in 5% of the examined 62 families [26]. Precise information on individuals with unilateral or bilateral affection was not given in this study, therefore, a comparison to the present study is only possible to a limited extent. Furthermore, it has to be pointed out that 6/8 (75%) solved cases had syndromic features additionally to bilateral kidney involvement and the diagnostic yield in syndromic cases was 18% (6/33). Also in the study by van der Ven et al., syndromic cases had a higher diagnostic yield than the total cohort (29% vs. 13%) [6]. Hence, our study, of course with limited sample size, encourages that syndromic features should actively be investigated in CAKUT cases with bilateral kidney involvement.

In this study, diagnostic yields were calculated on CAKUT phenotypic subgroups and individuals with bilateral kidney affection were grouped separately from individuals with unilateral kidney affection. This showed that all solved cases presented with bilateral kidney affection implying that, in individuals with bilateral kidney affection, comprehensive genetic testing should be considered, even in non-syndromic cases (2/8 of solved cases had bilateral kidney affection without reported extrarenal manifestations).

### Genetic diagnoses

All eight genetic diagnoses featured autosomal dominant syndromic monogenic CAKUT disorders (Table 2). However, only 6/8 (75%) solved cases had syndromic phenotypes as per medical records/referring clinicians. Reverse phenotyping of the two individuals without syndromic features after the molecular genetic result confirmed the isolated renal phenotype. And exactly these cases not featuring a syndromic phenotype carried a heterozygous disease-causing variant in *PAX2* (papillorenal syndrome, MIM #120330) and a heterozygous 1.2 Mb deletion on Chr17q12 encompassing *HNF1B* as the only disease-associated gene (17q12 deletion syndrome, MIM #614527). *PAX2* and *HNF1B* are the genes most frequently associated with autosomal dominant syndromic CAKUT accounting for 5–15% of cases in some studies [27]. An explanation for this observation could be that extrarenal manifestations can be subtle and missed by clinicians and that expressivity of CAKUT is highly variable and age-dependent (e.g., maturity-onset diabetes of the young and neuropsychiatric disorders in 17q12 deletion syndrome; the individual with 17q12 deletion syndrome was two years old at genetic testing and [reverse] phenotyping) [27]. Nonetheless, as mentioned above, our study makes the case that common syndromic monogenic CAKUT disorders could be present in bilateral kidney affection cases without apparent extrarenal manifestation, as phenotypes can be subtle and evolve over time.

Interestingly, in 2/8 solved cases, heterozygous disease-causing variants in *PBX1* could be identified, a fairly novel disease-associated gene at the time of ES [28]. This could illustrate an underestimated burden of *PBX1*-associated disease (CAKUTHED, MIM #617641) in monogenic CAKUT.

Two cases each carried disease-causing variants in *SALL1* and *EYA1*, both well-known autosomal dominant syndromic monogenic CAKUT genes (Townes-Brocks syndrome 1, MIM #107480; and branchiootorenal syndrome 1, MIM #113650) [27].

### Limitations

Although these are interesting observations, it has to be pointed out that this study has a small sample size. Therefore, and to confirm the findings, future larger studies should focus on diagnostic yields in phenotypic subgroups of CAKUT. This might allow the development of a clinical score for genetic testing in CAKUT in order to optimize clinical management.

A further limitation is the uneven distribution between our cases with unilateral/bilateral kidney affection (64/86; 74%) and isolated urine transport affection (22/86; 26%). The observed discrepancy between these two groups can probably be explained by the small study size and could change in a larger cohort.

For CAKUT cohorts, consanguinity of the parents is known to result in a higher diagnostic yield [13]. In our CAKUT cohort, however, this association was not present. This could refer to the overall limited number of cases with reported parental consanguinity (7/86, 8%) within this study.

## Conclusion

In this study, all solved cases had bilateral kidney involvement and 75% had syndromic disease showing this phenotypic presentation is a strong sign for an underlying hereditary cause in individuals with CAKUT. In future studies, a larger cohort of individuals with CAKUT is needed to develop a clinical score in order to increase the diagnostic yield in individuals with a CAKUT phenotype as molecular genetic diagnosis with ES can help to improve clinical management of individuals with CAKUT.

## Supplementary information

Supplementary Material

## Data Availability

The data that support the findings of this study are available on request from the corresponding author.
