# Supplementary material for: Exome sequencing in individuals with congenital anomalies of the kidney and urinary tract (CAKUT): a single-center experience
Source: Eur J Hum Genet. 2023 Mar 16;31(6):674–80. doi: 10.1038/s41431-023-01331-x (PMC10250376; doi:10.1038/s41431-023-01331-x)
Supplement: Supplementary file 1 — Supplementary Material [file 41431_2023_1331_MOESM1_ESM.docx]

**Supplementary Material**

**Exome sequencing in individuals with congenital anomalies of the kidney and urinary tract (CAKUT): a single-center experience**

Korbinian M. Riedhammer^1,2^, Jasmina Ćomić^1,2^, Velibor Tasic^3^, Jovana Putnik^4^, Nora Abazi-Emini^3^, Aleksandra Paripovic^4^, Natasa Stajic^4^, Thomas Meitinger^1^, Valbona Nushi-Stavileci^5^, Riccardo Berutti^1^, Matthias C. Braunisch^2^, Julia Hoefele^1^ and on behalf of the European Society for Paediatric Nephrology Working Group on Congenital Anomalies of the Kidney and Urinary Tract

**Supplementary Table 1:** **Phenotypic characteristics of 78 genetically unsolved index cases.** Complex CAKUT is defined as several CAKUT manifestations in an individual. CAKUT, congenital anomalies of the kidney and urinary tract;

| ID | Sex  (m= male;  f= female) | Non-Finnish European descent  (0= no;  1= yes;  2= unknown) | Core phenotype | Complex CAKUT (0= no;  1= yes) | Syndromic CAKUT  (0= no;  1= yes) | Reported family history  (0= no;  1= yes) | Reported parental consanguinity  (0= not reported;  1= reported) |
| --- | --- | --- | --- | --- | --- | --- | --- |
| HN-F67-II-1 | m | 1 | Bilateral renal dysplasia, left vesicoureteral reflux I-II | 1 | 0 | 0 | 0 |
| HN-F206-II-1 | f | 1 | Polycystic renal dysplasia, dolichocephaly | 0 | 1 | 0 | 0 |
| HN-F74-II-1 | m | 0 | Vesicoureteral reflux, left dysplastic kidney | 1 | 0 | 0 | 1 |
| HN-F526-II-1 | m | 1 | Bilateral renal dysplasia, type I diabetes | 1 | 1 | 0 | 0 |
| HN-F241-II-1 | f | 0 | Right duplex kidney, right hydronephrosis, short stature with dysostosis multiplex, developmental delay | 0 | 1 | 0 | 1 |
| HN-F16-II-2 | m | 0 | Bilateral hydronephrosis IV, hypertrophic cardiomyopathy | 0 | 1 | 0 | 0 |
| HN-F17-II-1 | m | 1 | Bilateral renal cystic dysplasia with obstructive uropathy, patent foramen ovale, lymphangiomatosis | 0 | 1 | 0 | 0 |
| HN-F18-II-3 | m | 1 | Bilateral renal agenesis, facial dysmorphies | 0 | 1 | 0 | 0 |
| HN-F183-II-1 | f | 0 | Small echogenic kidneys | 0 | 0 | 0 | 0 |
| HN-F193-II-2 | m | 1 | Bilateral renal dysplasia | 0 | 0 | 0 | 0 |
| HN-F328-II-1 | m | 1 | Left renal hypoplasia | 0 | 0 | 1 | 0 |
| HN-F522-II-7 | m | 0 | Left renal agenesis | 0 | 0 | 0 | 0 |
| HN-F71-III-2 | m | 0 | Bilateral renal hypoplasia, developmental delay, facial dysmorphies | 0 | 1 | 1 | 1 |
| HN-F539-II-1 | m | 1 | Bilateral renal dysplasia, imperforate anus | 0 | 1 | 0 | 0 |
| HN-F510-II-1 | m | 0 | Left renal agenesis, right renal dysplasia, cryptorchidism | 1 | 1 | 0 | 1 |
| HN-F538-II-2 | m | 0 | Bilateral renal agenesis | 0 | 0 | 0 | 0 |
| HN-F12-II-1 | f | 1 | Bilateral vesicoureteral reflux | 0 | 0 | 0 | 0 |
| HN-F189-II-1 | m | 0 | Bilateral renal dysplasia | 0 | 0 | 1 | 1 |
| HN-F215-III-1 | m | 1 | Renal dysgenesis | 0 | 0 | 1 | 0 |
| HN-F220-III-2 | f | 1 | Left renal aplasia, right renal dysplasia | 1 | 0 | 0 | 0 |
| HN-F221-III-5 | m | 1 | Hypoplastic solitary kidney | 0 | 0 | 0 | 0 |
| HN-F255-II-1 | m | 1 | Bilateral renal dysplasia, bilateral posterior urethral valves | 1 | 0 | 0 | 0 |
| HN-F59-II-3 | f | 1 | Bilateral multicystic dysplastic kidneys, tetralogy of Fallot | 0 | 1 | 0 | 0 |
| HN-F537-II-1 | m | 1 | Left renal agenesis, right hydronephrosis III, facial dysmorphies | 1 | 1 | 0 | 0 |
| HN-F14-II-1 | f | 0 | Right renal agenesis, left ectopic cystic kidney, left rocker bottom foot, left clinodactyly digitus V, facial dysmorphies | 1 | 1 | 0 | 0 |
| HN-F515-II-1 | f | 1 | Right duplex kidney, bilateral hearing impairment, muscular hypotonia | 0 | 1 | 0 | 0 |
| HN-F524-II-1 | f | 1 | Bilateral renal dysplasia, short stature | 0 | 1 | 0 | 0 |
| HN-F346-II-1 | m | 1 | Right renal cystic dysplasia | 0 | 0 | 0 | 0 |
| HN-F348-II-1 | m | 1 | Left ureteropelvic junction obstruction | 0 | 0 | 1 | 0 |
| HN-F349-II-1 | m | 1 | Bilateral vesicoureteral reflux V | 0 | 0 | 0 | 0 |
| HN-F351-II-1 | m | 1 | Bilateral vesicoureteral reflux V | 0 | 0 | 0 | 0 |
| HN-F354-II-1 | f | 1 | Left renal cystic dysplasia | 0 | 0 | 1 | 0 |
| HN-F357-II-1 | f | 1 | Bilateral vesicoureteral reflux III, right duplex kidney | 1 | 0 | 1 | 0 |
| HN-F358-II-1 | m | 1 | Right renal agenesis, uterine agenesis | 1 | 1 | 0 | 0 |
| HN-F362-II-1 | m | 1 | Left duplex kidney | 0 | 0 | 1 | 0 |
| HN-F527-II-1 | m | 1 | Unilateral hydroureteronephrosis, cardiomyopathy, short stature | 0 | 1 | 0 | 0 |
| HN-F383-II-1 | f | 1 | Right duplex kidney, bilateral vesicoureteral reflux III | 1 | 0 | 0 | 0 |
| HN-F406-II-1 | m | 1 | Left renal cystic dysplasia | 0 | 0 | 0 | 0 |
| HN-F417-II-1 | f | 1 | Right renal dysplasia, ectopic ureter (terminating at the urethra) | 1 | 0 | 0 | 0 |
| HN-F429-II-1 | m | 1 | Bilateral vesicoureteral reflux (left IV, right III) | 0 | 0 | 0 | 0 |
| HN-F435-II-1 | m | 1 | Bilateral megaureter | 0 | 0 | 0 | 0 |
| HN-F443-II-1 | f | 1 | Right renal agenesis | 0 | 0 | 0 | 0 |
| HN-F444-II-1 | m | 1 | Bilateral vesicoureteral reflux IV | 0 | 0 | 0 | 0 |
| HN-F446-II-1 | m | 1 | Left renal cystic dysplasia, bilateral hydronephrosis | 1 | 0 | 0 | 0 |
| HN-F449-II-1 | m | 1 | Left renal agenesis | 0 | 0 | 0 | 0 |
| HN-F451-II-1 | f | 1 | Right renal malrotation, ureteropelvic junction stenosis | 1 | 0 | 0 | 0 |
| HN-F454-II-2 | m | 1 | Right renal malrotation, hypospadias, mental retardation | 1 | 1 | 0 | 0 |
| HN-F480-II-1 | f | 1 | Left renal dysplasia | 0 | 0 | 0 | 0 |
| HN-F555-II-1 | f | 1 | Right renal hypoplasia, ectopic left kidney, developmental delay | 1 | 1 | 0 | 0 |
| HN-F559-II-1 | m | 1 | Left renal agenesis, cleft lip and palate | 0 | 1 | 0 | 0 |
| HN-F562-II-1 | f | 1 | Left renal cystic dysplasia, tetralogy of Fallot | 0 | 1 | 0 | 0 |
| HN-F563-II-1 | m | 1 | Bilateral vesicoureteral reflux IV | 0 | 0 | 0 | 0 |
| HN-F564-II-1 | m | 1 | Bilateral vesicoureteral reflux (left IV, right III), left hydronephrosis, ureteropelvic junction obstruction | 1 | 0 | 0 | 0 |
| HN-F565-II-1 | m | 1 | Bilateral vesicoureteral reflux IV | 0 | 0 | 0 | 0 |
| HN-F566-II-1 | m | 1 | Bilateral vesicoureteral reflux IV | 0 | 0 | 0 | 0 |
| HN-F569-II-1 | m | 1 | Right solitary kidney (prenatally: left cystic dysplastic kidney), left ureterocele | 1 | 0 | 0 | 0 |
| HN-F590-II-1 | f | 1 | Left renal agenesis | 0 | 0 | 0 | 0 |
| HN-F593-II-1 | m | 1 | Right renal cystic dysplasia | 0 | 0 | 0 | 0 |
| HN-F605-II-1 | f | 1 | Left renal cystic dysplasia | 0 | 0 | 0 | 0 |
| HN-F606-II-1 | m | 1 | Left renal cystic dysplasia | 0 | 0 | 0 | 0 |
| HN-F608-II-1 | F | 1 | Left renal agenesis | 0 | 0 | 0 | 0 |
| HN-F622-II-1 | f | 1 | Right duplicated ureter, left vesicoureteral reflux III | 1 | 0 | 0 | 0 |
| HN-F623-II-1 | m | 1 | Left renal agenesis, right ectopic kidney | 1 | 0 | 0 | 0 |
| HN-F625-II-1 | m | 1 | Left renal agenesis | 0 | 0 | 0 | 0 |
| HN-F626-II-1 | m | 1 | Left renal agenesis | 0 | 0 | 0 | 0 |
| HN-F627-II-2 | m | 1 | Hypospadias | 0 | 0 | 1 | 0 |
| HN-F640-II-1 | m | 1 | Ureteropelvic junction obstruction, ventricular septal defect | 0 | 1 | 0 | 0 |
| HN-F646-II-1 | m | 0 | Bilateral renal dysplasia, mental retardation, failure to thrive, microcephaly | 0 | 1 | 0 | 1 |
| HN-F647-II-1 | f | 0 | Bilateral renal dysplasia, mental retardation, hearing impairment, microcephaly | 0 | 1 | 0 | 1 |
| HN-F657-II-1 | f | 1 | Left renal agenesis, double uterus | 1 | 1 | 0 | 0 |
| HN-F665-II-1 | m | 1 | Bilateral renal cystic dysplasia, bilateral hydronephrosis, seizures, patent foramen ovale, mild muscular hypotonia | 1 | 1 | 0 | 0 |
| HN-F514-II-2 | m | 1 | Lower urinary tract obstruction | 0 | 0 | 1 | 0 |
| HN-F379-II-1 | m | 1 | Left renal cystic dysplasia | 0 | 0 | 1 | 0 |
| HN-F312-II-3 | m | 1 | Right renal hypoplasia | 0 | 0 | 0 | 0 |
| HN-F80-II-1 | f | 1 | Bilateral renal hypoplasia, visual impairment, short stature | 1 | 1 | 0 | 0 |
| HN-F347-II-1 | m | 1 | Right renal cystic dysplasia, Congenital ptosis on the right, epicanthus, mild retrognathia, left inguinal hernia, Eye malformations | 0 | 1 | 0 | 0 |
| HN-F598-II-1 | m | 1 | Right renal cystic dysplasia | 0 | 0 | 0 | 0 |
| HN-F602-II-1 | m |  | Right ectopic and dysplastic kidney | 0 | 0 | 1 | 0 |

**Supplementary Table 2. List of homozygous stretches** **in exome sequencing data with homozygous rare variants (MAF<1.0%) within these stretches in consanguineous cases (all unsolved). Only variants in OMIM-listed (https://www.omim.org/) disease-associated genes reported.**

| Individual | Regions of homozygosity (UCSC) | Chromosomal position | Gene | Nucleotide change (HGVSc) | Aminoacid change (HGVSp) | OMIM |
| --- | --- | --- | --- | --- | --- | --- |
| HN-F74-II-1 | chr1:159825061-161495476 |  |  |  |  |  |
|  | chr1:161645259-168545863 |  |  |  |  |  |
|  | chr1:169080837-170501384 |  |  |  |  |  |
|  | chr1:175348723-201178469 |  |  |  |  |  |
|  | chr1:201181217-209779804 |  |  |  |  |  |
|  | chr1:229738170-231903009 |  |  |  |  |  |
|  | chr2:119735251-130941064 |  |  |  |  |  |
|  | chr2:131100496-133174763 |  |  |  |  |  |
|  | chr2:234590974-234637802 |  |  |  |  |  |
|  | chr3:42597217-44763036 |  |  |  |  |  |
|  | chr4:128554404-133367587 |  |  |  |  |  |
|  | chr8:26944600-37333588 |  |  |  |  |  |
|  | chr8:37968218-38677931 |  |  |  |  |  |
|  | chr10:27375368-28023746 |  |  |  |  |  |
|  | chr10:70167004-70856851 |  |  |  |  |  |
|  | chr10:95432968-96954510 |  |  |  |  |  |
|  | chr11:63885769-64110450 |  |  |  |  |  |
|  | chr12:49054491-50344975 |  |  |  |  |  |
|  | chr12:65881702-72666498 |  |  |  |  |  |
|  | chr12:75435999-79611373 |  |  |  |  |  |
|  | chr12:125324570-126932623 |  |  |  |  |  |
|  | chr15:25925195-31776003 |  |  |  |  |  |
|  | chr17:5418799-6014175 |  |  |  |  |  |
|  | chr17:15341022-18344665 |  |  |  |  |  |
|  | chr17:18574872-20355057 | chr17:18063314-18063314 | *MYO15A* | NM_016239.4:c.9369C>A | p.Asp3123Glu | 602666 |
|  | chr17:21731041-32612893 |  |  |  |  |  |
|  | chr18:658064-2885117 |  |  |  |  |  |
|  | chr18:77439532-77927027 |  |  |  |  |  |
|  | chr19:37945601-38994740 |  |  |  |  |  |
|  | chr19:52376119-52393983 |  |  |  |  |  |
| HN-F241-II-1 | chr1:25678238-26596079 |  |  |  |  |  |
|  | chr1:26608883-31186386 |  |  |  |  |  |
|  | chr1:171707272 196227525 |  |  |  |  |  |
|  | chr1:230451978-249230767 |  |  |  |  |  |
|  | chr2:15415525-17884373 |  |  |  |  |  |
|  | chr2:38179395-54494369 |  |  |  |  |  |
|  | chr2:102782433-114357349 |  |  |  |  |  |
|  | chr2:141113798-155265622 |  |  |  |  |  |
|  | chr2:179395958-179501350 |  |  |  |  |  |
|  | chr2:189862097-208811096 |  |  |  |  |  |
|  | chr2:238234285-240912982 |  |  |  |  |  |
|  | chr2:241403965-242836534 |  |  |  |  |  |
|  | chr3:155314034-171830204 |  |  |  |  |  |
|  | chr3:172428508-194877354 |  |  |  |  |  |
|  | chr4:1388952-8217793 |  |  |  |  |  |
|  | chr4:38776107-62383314 |  |  |  |  |  |
|  | chr4:129012181-139964054 |  |  |  |  |  |
|  | chr5:63905068-68464233 |  |  |  |  |  |
|  | chr6:17493690-22136878 |  |  |  |  |  |
|  | chr6:35960390-42571345 | chr6:38913287-38913287 | *DNAH8* | NM_001206927.2:c.12052C>T | p.Arg4018Cys | 603337 |
|  | chr6:44107365-57244733 |  |  |  |  |  |
|  | chr6:62526443-90448091 |  |  |  |  |  |
|  | chr6:131540726-142384081 |  |  |  |  |  |
|  | chr7:21640361-39500351 |  |  |  |  |  |
|  | chr7:149503843-149744834 |  |  |  |  |  |
|  | chr9:5077517-6990363 |  |  |  |  |  |
|  | chr9:70993067-77230476 |  |  |  |  |  |
|  | chr9:84200599-88269946 |  |  |  |  |  |
|  | chr10:7244242-9449825 |  |  |  |  |  |
|  | chr10:11356160-46999189 |  |  |  |  |  |
|  | chr10:49392575-55588136 |  |  |  |  |  |
|  | chr10:73537358-81342368 |  |  |  |  |  |
|  | chr10:81471548-85908687 |  |  |  |  |  |
|  | chr10:96609568-97006854 |  |  |  |  |  |
|  | chr11:51459390-57166996 | chr11:35250873-35250873 | *CD44* | NM_000610.4:c.2222G>A | p.Gly741Glu | 107269 |
|  | chr11:57799371-58919812 |  |  |  |  |  |
|  | chr11:76207633-92257702 |  |  |  |  |  |
|  | chr12:862989-8048012 |  |  |  |  |  |
|  | chr12:10233652-11420374 |  |  |  |  |  |
|  | chr12:12240199-27461983 |  |  |  |  |  |
|  | chr12:122186317-132396602 | chr12:122437794-122437794 | *WDR66* | NM_144668.6:c.3179C>A | p.Ser1060Tyr | 618146 |
|  | chr12:114296739-122064784 |  |  |  |  |  |
|  | chr12:132862976-133810702 |  |  |  |  |  |
|  | chr13:33590851-38138688 |  |  |  |  |  |
|  | chr13:99448441-100199223 |  |  |  |  |  |
|  | chr14:67675224-68008457 |  |  |  |  |  |
|  | chr15:40645347-41146580 |  |  |  |  |  |
|  | chr15:51749726-52897477 |  |  |  |  |  |
|  | chr15:89436244-90308025 |  |  |  |  |  |
|  | chr15:90333980-93198670 |  |  |  |  |  |
|  | chr16:67254-593276 |  |  |  |  |  |
|  | chr16:712127-2485895 |  |  |  |  |  |
|  | chr16:2747857-4386813 |  |  |  |  |  |
|  | chr16:4432029-5037522 |  |  |  |  |  |
|  | chr17:13446924-18528622 | chr17:17716004-17716004 | *SREBF1* | NM_004176.4:c.3376C>T | p.Arg1126Trp | 184756 |
|  | chr17:18574872-21194629 |  |  |  |  |  |
|  | chr17:25630388-35295229 |  |  |  |  |  |
|  | chr17:63533768-72348740 |  |  |  |  |  |
|  | chr18:5196992-7023370 |  |  |  |  |  |
|  | chr19:307037-367312 |  |  |  |  |  |
|  | chr19:1047002-1753588 |  |  |  |  |  |
|  | chr19:3778219-4508944 |  |  |  |  |  |
|  | chr19:4847713-5787224 |  |  |  |  |  |
|  | chr19:5844537-8111792 |  |  |  |  |  |
|  | chr19:8122737-16582936 | chr19:8808573-8808573 | *ACTL9* | NM_178525.5:c.479A>G | p.Glu160Gly | 619251 |
|  | chr19:16601194-17488140 |  |  |  |  |  |
|  | chr19:49513273-51128660 |  |  |  |  |  |
|  | chr20:76962-2633233 |  |  |  |  |  |
|  | chr20:5902853-6195663 |  |  |  |  |  |
|  | chr20:13029764-42744801 |  |  |  |  |  |
|  | chr20:57266592-59827663 |  |  |  |  |  |
|  | chr22:35463179-38689983 |  |  |  |  |  |
|  | chr22:38875549-43739203 |  |  |  |  |  |
|  | chr22:47309290-50017233 |  |  |  |  |  |
| HN-F71-III-2 | chr1:2303371-6133700 |  |  |  |  |  |
|  | chr1:38155699-40092088 |  |  |  |  |  |
|  | chr1:41101572-57111168 |  |  |  |  |  |
|  | chr1:221509161-228108931 |  |  |  |  |  |
|  | chr2:26203678-46231524 |  |  |  |  |  |
|  | chr2:86107987-90008207 |  |  |  |  |  |
|  | chr2:92031762-97860528 |  |  |  |  |  |
|  | chr2:98164084-105472054 |  |  |  |  |  |
|  | chr2:138169158-141473504 |  |  |  |  |  |
|  | chr3:133108978-174581887 | chr3:138664795-138664795 | *FOXL2* | NM_023067.4:c.770C>A | p.Pro257Gln | 605597 |
|  | chr3:176752264-185879295 |  |  |  |  |  |
|  | chr4:38774785-62383314 |  |  |  |  |  |
|  | chr5:162890953-177099273 |  |  |  |  |  |
|  | chr5:177155848-178949559 |  |  |  |  |  |
|  | chr5:179021982-180486840 |  |  |  |  |  |
|  | chr6:24174955-29910285 |  |  |  |  |  |
|  | chr6:29977145-31238727 |  |  |  |  |  |
|  | chr6:31324516-32489638 | chr6:31833725-31833725 chr6:31833726-31833726 | *SLC44A4 SLC44A4* | NM_025257.3:c.1412C>T NM_025257.3:c.1411T>G | p.Ser471Phe p.Ser471Ala | 606107 606107 |
|  | chr6:32627923-45880310 |  |  |  |  |  |
|  | chr7:1733192-4780513 |  |  |  |  |  |
|  | chr7:99751017-100274062 |  |  |  |  |  |
|  | chr8:107754583-116635941 |  |  |  |  |  |
|  | chr9:79835177-80409344 |  |  |  |  |  |
|  | chr12:117217141-119563063 |  |  |  |  |  |
|  | chr15:41245676-41828570 |  |  |  |  |  |
|  | chr15:78857939-79083590 |  |  |  |  |  |
|  | chr16:14014240-16360454 |  |  |  |  |  |
|  | chr16:17221755-28354089 |  |  |  |  |  |
|  | chr16:28780994-32893879 | chr16:28857574-28857574 | *TUFM* | NM_003321.5:c.17C>T | p.Ala6Val | 602389 |
|  | chr16:49432926-54967095 |  |  |  |  |  |
|  | chr16:84203612-84270380 |  |  |  |  |  |
|  | chr17:11459012-12569441 |  |  |  |  |  |
|  | chr18:60630531-70209320 |  |  |  |  |  |
|  | chr19:10073324-11314825 |  |  |  |  |  |
|  | chr19:11319978-14752324 |  |  |  |  |  |
|  | chr19:45126994-46800432 |  |  |  |  |  |
|  | chr19:55086775-55146803 |  |  |  |  |  |
|  | chr20:61299525-62737317 |  |  |  |  |  |
|  | chr22:41604353-45680823 |  |  |  |  |  |
|  | chr22:45732328-49661314 |  |  |  |  |  |
|  | chr22:50017234-51183254 |  |  |  |  |  |
| HN-F510-II-1 | chr1:38185647-38272659 |  |  |  |  |  |
|  | chr1:65270549-67195183 |  |  |  |  |  |
|  | chr1:150122493-152079822 |  |  |  |  |  |
|  | chr1:248343945-249110905 |  |  |  |  |  |
|  | chr2:127808532-128385339 |  |  |  |  |  |
|  | chr3:38009700-38134437 |  |  |  |  |  |
|  | chr4:103534560-104510765 |  |  |  |  |  |
|  | chr4:128554404-139093227 |  |  |  |  |  |
|  | chr5:89769647-108171482 |  |  |  |  |  |
|  | chr6:36653477-39282035 |  |  |  |  |  |
|  | chr7:91630620-93540347 |  |  |  |  |  |
|  | chr7:140255657-142124169 |  |  |  |  |  |
|  | chr8:119964052-120940651 |  |  |  |  |  |
|  | chr9:72003036-74300639 |  |  |  |  |  |
|  | chr10:85944313-87489414 |  |  |  |  |  |
|  | chr10:96014622-96796860 |  |  |  |  |  |
|  | chr11:68133035-68305493 |  |  |  |  |  |
|  | chr11:118037813-123065719 |  |  |  |  |  |
|  | chr12:101732655-102295217 |  |  |  |  |  |
|  | chr14:105309256-106054427 |  |  |  |  |  |
|  | chr14:106110137-106733519 |  |  |  |  |  |
|  | chr15:34435255-34640217 |  |  |  |  |  |
|  | chr19:7153006-8319454 |  |  |  |  |  |
|  | chr19:36486509-37039068 |  |  |  |  |  |
|  | chr19:52223121-52852522 |  |  |  |  |  |
| HN-F189-II-1 | chr7:91630620-92734450 |  |  |  |  |  |
|  | chr9:139252495-139564473 |  |  |  |  |  |
|  | chr9:140002989-140242643 |  |  |  |  |  |
|  | chr10:49939139-50121453 |  |  |  |  |  |
|  | chr12:2659082-2964683 |  |  |  |  |  |
|  | chr19:37441111-37854206 |  |  |  |  |  |
|  | chr22:46627603-46929691 |  |  |  |  |  |
| HN-F646-II-1 | chr1:99150635-144193366 |  |  |  |  |  |
|  | chr1:145510376-176992552 |  |  |  |  |  |
|  | chr1:220145371-222721287 |  |  |  |  |  |
|  | chr1:222802006-223716251 | chr1:229600517-229600517 | *NUP133* | NM_018230.3:c.2405C>A | p.Thr802Asn | 607613 |
|  | chr1:223799857-231664606 |  |  |  |  |  |
|  | chr1:247242027-249110905 |  |  |  |  |  |
|  | chr2:75915035-97166203 |  |  |  |  |  |
|  | chr2:128079806-129025704 |  |  |  |  |  |
|  | chr2:130899804-131672949 |  |  |  |  |  |
|  | chr2:135629927-136467118 |  |  |  |  |  |
|  | chr2:190561060-192701616 |  |  |  |  |  |
|  | chr2:218604247-222290901 |  |  |  |  |  |
|  | chr2:234621787-234652184 |  |  |  |  |  |
|  | chr2:235951819-240500172 |  |  |  |  |  |
|  | chr3:190991439-194842876 |  |  |  |  |  |
|  | chr3:195723937-197581146 |  |  |  |  |  |
|  | chr4:110603784-144801661 |  |  |  |  |  |
|  | chr4:144915328-154178696 |  |  |  |  |  |
|  | chr4:185552345-185655170 |  |  |  |  |  |
|  | chr5:822109-3600333 |  |  |  |  |  |
|  | chr6:32230354-32369487 |  |  |  |  |  |
|  | chr6:32627923-32709308 |  |  |  |  |  |
|  | chr6:44240193-124603377 |  |  |  |  |  |
|  | chr6:166307718-167271710 |  |  |  |  |  |
|  | chr7:31439-772173 |  |  |  |  |  |
|  | chr7:881668-2582152 |  |  |  |  |  |
|  | chr7:2636689-5396954 |  |  |  |  |  |
|  | chr7:5415874-24324807 |  |  |  |  |  |
|  | chr7:28857606-100547484 | chr7:31918726-31918726 chr7:70163580-70163582 chr7:91732040-91732040 chr7:99704392-99704417 chr7:21920443-21920443 | *PDE1C AUTS2 AKAP9 AP4M1 DNAH11* | NM_001191057.4:c.308G>A NM_015570.4:c.718_720del NM_005751.4:c.11230G>T NM_004722.4:c.1257_1282del NM_001277115.2:c.12319C>G | p.Arg103Gln p.Phe240del p.Gly3744Trp p.Val421AlafsTer98 p.Pro4107Ala | 602987 607270 604001 602296 603339 |
|  | chr7:100841389-103389953 |  |  |  |  |  |
|  | chr7:131191536-132070053 |  |  |  |  |  |
|  | chr7:150919383-157151233 |  |  |  |  |  |
|  | chr7:157873975-158851803 |  |  |  |  |  |
|  | chr9:117934-6986290 |  |  |  |  |  |
|  | chr9:136081391-136268037 |  |  |  |  |  |
|  | chr11:28134974-69924225 |  |  |  |  |  |
|  | chr12:20766559-40881963 |  |  |  |  |  |
|  | chr12:40893496-50744093 |  |  |  |  |  |
|  | chr12:50899153-55759877 |  |  |  |  |  |
|  | chr12:55945119-72388373 |  |  |  |  |  |
|  | chr12:74932159-82617278 |  |  |  |  |  |
|  | chr13:36744910-37007039 |  |  |  |  |  |
|  | chr14:31355096-34985644 |  |  |  |  |  |
|  | chr15:50754339-51502843 |  |  |  |  |  |
|  | chr15:79024268-79217689 |  |  |  |  |  |
|  | chr16:601892-726880 |  |  |  |  |  |
|  | chr16:49412531-65397281 |  |  |  |  |  |
|  | chr16:77769369-82673046 |  |  |  |  |  |
|  | chr17:37349710-39471762 |  |  |  |  |  |
|  | chr17:39506659-55917352 |  |  |  |  |  |
|  | chr19:45121424-48282077 |  |  |  |  |  |
|  | chr19:48382480-48977018 |  |  |  |  |  |
|  | chr20:23345844-23729721 |  |  |  |  |  |
|  | chr20:60966318-61912714 |  |  |  |  |  |
|  | chr22:50014330-51183254 |  |  |  |  |  |
| HN-F647-II-1 | chr1:236700807-236882109 |  |  |  |  |  |
|  | chr3:45979877-46399797 |  |  |  |  |  |
|  | chr3:121351338-121976252 |  |  |  |  |  |
|  | chr5:136961400-140683264 |  |  |  |  |  |
|  | chr10:75407290-75673100 |  |  |  |  |  |
|  | chr10:100147060-100190263 |  |  |  |  |  |
|  | chr12:121131891-121592688 |  |  |  |  |  |
|  | chr16:71660310-72050884 |  |  |  |  |  |

**Supplementary Table 3. List of rare *de novo* variants (MAF<0.1%; CADD [https://cadd.gs.washington.edu/] >15) and rare homozygous/compound-heterozygous/hemizygous variants (MAF<1.0%) in unsolved trio exome sequencing cases. Only variants in OMIM-listed (https://www.omim.org/) disease-associated genes reported.**

| Individual | Gene | Inheritance | Chromosomal position | Nucleotide change (HGVSc) | Aminoacid change (HGVSp) | Zygosity | OMIM |
| --- | --- | --- | --- | --- | --- | --- | --- |
| HN-F16-II-2 | *PLOD2* | *De-novo* | chr3:145803031-145803031 | NM_182943.3:c.1157A>G | p.Asp386Gly | heterozygous | 601865 |
| HN-F590-II-1 | *XPNPEP3* | *De-novo* | chr22:41320408-41320408 | NM_022098.4:c.1279G>A | p.Gly427Arg | heterozygous | 613553 |
| HN-F625-II-1 | *RP1L1* | *De-novo* | chr8:10469315-10469315 | NM_178857.6:c.2293G>T | p.Asp765Tyr | heterozygous | 608581 |
| HN-F626-II-1 | *CLCN5* | maternal | chrX:49851084-49851084 | NM_001127898.4:c.1114C>T | p.Arg372Cys | hemizygous | 300008 |
| HN-F646-II-1 | *NUP133* | maternal and paternal | chr1:229600517-229600517 | NM_018230.3:c.2405C>A | p.Thr802Asn | homozygous | 607613 |
